# Physical discipline as a normative childhood experience in Singapore

**DOI:** 10.1186/s13034-023-00632-9

**Published:** 2023-06-29

**Authors:** Mioko Sudo, Ying Qing Won, Winnie W. Y. Chau, Michael J. Meaney, Michelle Z. L Kee, Helen Chen, Johan Gunnar Eriksson, Fabian Yap, Anne Rifkin-Graboi, Henning Tiemeier, Peipei Setoh

**Affiliations:** 1 Psychology Division, School of Social Sciences, Nanyang Technological University, 48 Nanyang Ave, Singapore, 639818 Singapore; 2 Department of Psychology, National University of Singapore, 9 Arts Link, Singapore, 117570 Singapore; 3 Agency for Science, Technology and Research (ASTAR), 30 Medical Drive, Brenner Centre for Molecular Medicine, Singapore Institute for Clinical Sciences (SICS), Singapore, 117609 Singapore; 4 Sackler Program for Epigenetics & Psychobiology, Douglas Mental Health University Institute, McGill University, 6875 Boulevard LaSalle, Montréal, QC H4H 1R3 Canada; 5 Ludmer Centre for Neuroinformatics and Mental Health, Douglas Mental Health University Institute, McGill University, 6875 Boulevard LaSalle, Montréal, QC H4H 1R3 Canada; 6 Department of Psychological Medicine, KK Women’s and Children’s Hospital, 100 Bukit Timah Road, Singapore, 229899 Singapore; 7 Duke-NUS Medical School, 8 College Rd, Singapore, 169857 Singapore; 8 Department of Obstetrics and Gynaecology, Human Potential Translational Research Programme, Yong Loo Lin School of Medicine, National University of Singapore, 10 Medical Dr, Singapore, 117597 Singapore; 9 Human Potential Translational Research Programme, Yong Loo Lin School of Medicine, National University of Singapore, 10 Medical Dr, Singapore, 117597 Singapore; 10 Department of General Practice and Primary Health Care, University of Helsinki, Tukholmankatu 8 B, 00290 Helsinki, Finland; 11 Public Health Research Program, Folkhälsan Research Center, Topeliuksenkatu 20, 00250 Helsinki, Finland; 12 Department of Paediatrics, KK Women’s and Children’s Hospital, 100 Bukit Timah Road, Singapore, 229899 Singapore; 13 Academic Medicine Department, Duke-NUS Medical School, 8 College Rd, Singapore, 169857 Singapore; 14 Lee Kong Chian School of Medicine, Nanyang Technological University, 59 Nanyang Dr, Singapore, 636921 Singapore; 15 Centre for Research in Child Development, National Institute of Education, Nanyang Technological University, 1 Nanyang Walk, Singapore, 637616 Singapore; 16 Department of Social and Behavioral Science, Harvard T. H. Chan School of Public Health, Harvard University, 677 Huntington Ave, Boston, MA 02115 USA

**Keywords:** Physical, Corporal, Discipline, Singapore, Parenting

## Abstract

**Background:**

The cultural normativeness theory posits that specific parenting behaviors can be interpreted as displays of appropriate parenting in contexts where they are deemed normative. Previous studies suggest high acceptance of physical discipline in Singapore, where strict parenting could be interpreted as care for the child. However, there is a lack of studies on the local prevalence and implications of physical discipline. This study aimed to investigate the prevalence of Singaporean children experiencing parental physical discipline, longitudinal changes in this prevalence, and how exposure to physical discipline relates to children’s evaluation of their parents’ parenting.

**Methods:**

Participants were 710 children with parental reports of physical discipline at one or more assessments at ages 4.5, 6, 9, and 11 years in the Growing Up in Singapore Towards healthy Outcomes birth cohort study. Parental reports of physical discipline were obtained using the Parenting Styles and Dimensions Questionnaire or the Alabama Parenting Questionnaire across the four assessments. Child reports of their parents’ care and control were obtained using the Parental Bonding Instrument for Children at the age 9 assessment. Prevalence was specified as being exposed to at least one physical discipline at any frequency. A generalized linear mixed model was performed to examine whether children’s age predicted their exposure to physical discipline. Linear regression analyses were conducted to investigate whether children’s exposure to physical discipline predicted their evaluation of their parents’ parenting.

**Results:**

The prevalence of children experiencing at least one physical discipline was above 80% at all ages. There was a decrease in this prevalence from age 4.5 to 11 years (B = − 0.14, *SE* = 0.01, OR = 0.87, *p* < 0.001). The more frequent the paternal physical discipline children were exposed to, the more likely they were to report lower levels of care (B = − 1.74, *SE* = 0.66, *p* = 0.03) and higher levels of denial of psychological autonomy by fathers (B = 1.05, *SE* = 0.45, *p* = 0.04). Maternal physical discipline was not significantly associated with children’s evaluation of their mothers’ parenting (*p*s ≥ 0.53).

**Conclusions:**

Physical discipline was a common experience among our Singaporean sample, consistent with the notion that strict parenting could be regarded as a form of care. However, exposure to physical discipline did not translate to children reporting their parents as caring, with paternal physical discipline being negatively associated with children’s evaluations of paternal care.

**Supplementary Information:**

The online version contains supplementary material available at 10.1186/s13034-023-00632-9.

## Background

Physical discipline refers to the use of physical force to inflict bodily pain, but not injury, with the intention of correcting or controlling the child’s behavior [1]. Meta-analyses of the association between physical discipline and developmental outcomes have suggested that physical discipline is not beneficial to child development, but places children at risk for a wide range of negative consequences, including internalizing and externalizing problems, poor mental health, increased aggression, and low self-esteem [2–4]. Yet, the notion that physical discipline is inadvisable has not been universally endorsed, particularly among proponents of the ‘conditional corporal punishment perspective’ [5–7]. This perspective suggests that physical discipline, unless severe and frequent, could be beneficial as a back-up strategy to milder disciplinary tactics (e.g., time-out, privilege removal) when noncompliance persists [5–7]. Nonetheless, the great majority of the empirical literature to date suggests that physical discipline, even in mild forms (i.e., spanking), is harmful to children’s developmental outcomes [8–10]. The cultural normativeness theory also suggests that physical discipline may not be universally detrimental, but could be associated with less adverse child outcomes in cultures where physical discipline is normative and hence less likely to be interpreted as hostile or threatening by children [11]. Previous studies testing this theory, while limited in number, yield inconclusive findings. Few studies suggest that cultural normativeness can mitigate, but not fully eliminate, the negative impacts of physical discipline on child outcomes [12, 13], while few other studies find no support for the moderating role of cultural normativeness in the potential impacts of physical discipline. [9, 14]

A useful framework for understanding parenting across cultures is that there can be cross-cultural differences in form (e.g., behavior) and function (e.g., meaning attached to behavior) of parenting [15, 16]. In line with this framework, there is wide variability across countries in the prevalence of various forms of discipline (i.e., physical aggression, psychological aggression, nonviolent discipline) [17], and parents’ perception of the acceptability, efficacy, and necessity of certain forms of discipline [18]. The cultural normativeness theory posits that the function of parenting behaviors can differ by their normativeness, such that the parent and child may interpret specific behaviors, including harsh discipline, as appropriate displays of care in contexts where such behaviors are deemed acceptable [11]. For example, Chao [19] suggested that ‘training’ (*chiao shun*) and ‘governing’ (*guan*) of the child through strict control tend to be associated with care and concern for the child in Chinese cultures, whereas such practices could be interpreted as forms of hostility in European-American cultures. Further, firm control and governance of the child could be considered as parental responsibilities in Chinese cultures, such that not engaging in such practices would be considered negligent and uncaring [20]. The culture-specific function of strict parenting as showing care for the child has been suggested to have its roots in Confucian principles that elders are responsible for firmly disciplining and governing children to meet familial and societal goals, and that children must show filial piety, or loyalty and respect to elders [19, 21]. Singapore provides a unique sociocultural landscape that is at the crossroads of the East and West. Despite being an Asian country with the majority of the population (74.3%) comprised of ethnically Chinese individuals [22], Singapore has been heavily influenced by Western values due to its history as a British colony, rapid globalization since its independence, and use of English as the lingua franca [23]. In Eastern cultural contexts that are increasingly exposed to Western values, parents’ socialization goals could transition from focusing on interdependence-oriented values to embodying a combination of such values with more independence-oriented values. For example, with regards to emotion socialization, parents may aim to foster their child’s ability to independently manage their emotions while maintaining interdependence with others [24, 25]. Nonetheless, parenting in Singapore has been suggested to have similarities with traditional Chinese parenting as described in previous literature, such that strict parenting could be endorsed as a form of care for the child and hence may not necessarily have negative connotations for children [23, 26–29].

In line with the idea that strict parenting could be endorsed in Singapore, previous research suggests high acceptance of caning (i.e., hitting with a rattan stick) among Singaporean adults. In a 1994 survey, 72% of Singaporean adults indicated that caning children was ‘sometimes’ or ‘always’ acceptable [30]. Caning was particularly considered acceptable under certain conditions, such as if the adult had good intentions, if it happened infrequently, if only the limbs or buttocks were affected, and if no permanent marks or injuries were left. Further, 72% of adults indicated that caning children ‘can be’ or is ‘not’ abuse, rather than unambiguously indicating that it ‘is’ abuse. This figure has not decreased over time, with 78% of Singaporean adults indicating that caning children is not definitively a form of abuse in a 2010 survey [31]. Slapping children on the face and shaking children hard are less accepted than caning but may still be accepted by a large proportion of adults in Singapore, with roughly half of the adults in the 1994 and 2010 surveys indicating that the practices are not definitively abusive [30, 31]. The two surveys also examined public perception of two more forms of physical aggression, tying children up and burning children, with results suggesting that these two practices were almost invariably considered unacceptable and abusive. The belief that physical discipline is typical and appropriate is a strong predictor of parents’ use of the practice [32–34]. Previous literature suggests wide acceptability of physical discipline (i.e., caning) and potential endorsement of strict parenting as a form of care in Singapore, but there is a lack of studies on the actual prevalence of children being physically disciplined by their parents.

It is also important to consider whether the prevalence of physical discipline in Singapore could differ by demographic characteristics, particularly child sex, parent sex, and parent ethnicity. Previous studies on prevalence of physical discipline have commonly reported that boys are more likely to be physically disciplined than girls, potentially due to gender expectations that boys require harsher discipline to correct their misbehavior, or higher parental expectations for boys to become future leaders within the household and society [35–37]. Another commonly reported finding is that mothers are more likely than fathers to use physical discipline, potentially due to spending more time with their child as the main caregiver, and hence having more opportunities to become aware of and to correct their misbehavior [38, 39]. These differential patterns of prevalence of physical discipline by child and parent sex have also been found in a study conducted in mainland China [40]. Finally, Singapore is a multi-ethnic society comprised of 74.3% Chinese, 13.5% Malay, 9.0% Indian, and 3.2% other-ethnicity individuals [22]. The characteristics of Chinese parenting reported in previous literature, such as strict control being associated with care, are likely to be most relevant to the ethnically Chinese parents, and potentially less relevant to the ethnically Malay and Indian parents, in Singapore. For example, an observational study in Singapore on mother–child interactions during a buffet meal found that Chinese mothers were more likely than Malay and Indian mothers to question their child’s food choices, consistent with the notion that Chinese parents engage in governance and training of their child. [41]

The prevalence of physical discipline is not constant across childhood. Parents could become less reliant on physical discipline as their children enter middle childhood and develop the cognitive maturity to understand reasoning, recognize the consequences of their misbehavior, and control their own behavior [42–44]. Further, the parent–child relation may gradually transform from one that revolves around parental authority to one that involves mutual decision making between the parent and child [43]. In accordance with such ideas, previous studies in non-Asian countries (e.g., United States, Canada, Finland, Sweden, Colombia) have found that exposure to physical discipline tends to be more prevalent among preschool-aged children than children in their middle childhood [3, 35, 39, 45–47]. Further, studies in the United States providing fine-grained reports of prevalence rates by each year of child age suggest that exposure to physical discipline peaks in and gradually declines after the preschool years [35, 39]. Interestingly, one study conducted in mainland China found partially discrepant patterns such that parents’ use of coercive discipline (including physical discipline) decreased by late childhood, but peaked at age 7 when children had started formal schooling [40]. The authors postulated that Chinese parents could become more motivated to use coercive discipline at this time, due to their increasing expectations for children to achieve academic and social goals as they enter school, and their increasing intolerance for behavioral problems as children are often considered as being capable of moral understanding (*dongshi*) at age 6 or 7.

These postulations are not sufficient to make predictions for trajectories of physical discipline over childhood in Singapore, but nonetheless highlight the importance of examining whether the commonly reported decline in physical discipline after the preschool years can be replicated in Singapore’s unique cultural context. First, filial piety is a highly valued construct in Singapore and an integral part of parent–child relations even in adulthood [48]. However, in a cultural context that is influenced by a combination of interdependence-oriented Eastern values and independence-oriented Western values, there could be some tension between valuing the exertion of parental authority and recognizing the merits of parenting approaches that prioritize children’s sense of autonomy [23]. Hence, it is unclear to what extent parent–child relations would shift towards prioritizing mutual decision making, and correspondingly less parental coercion, over childhood. Second, Singaporean parents place a high priority on their children’s education [49, 50]. Academic pressures could rise from early to middle childhood in Singapore as children prepare for the Primary School Leaving Examination, a high-stakes national exam taken at age 12 that not only determines which secondary school children can go to, but is also often considered to affect long-term success [51, 52]. It is important to consider how exposure to strict parenting could change during this time, particularly since parenting in Singapore has been discussed in light of previous literature on Chinese parenting, where ‘training’ (*chiao shun*) of the child through strict control often focuses on driving them to achieve in academics [19].

Parental physical discipline has been associated with decreased quality of parent–child relationships, potentially due to children interpreting physical discipline as a form of parental rejection and generalizing the negative affect (e.g., fear, anger, anxiety) evoked by physical discipline to their parents [3, 4, 53]. Further, a recent study found that young adults in Israel who were physically disciplined by their parents as a child evaluated the parenting they received less positively (i.e., less loving, meaner, and less good as other parents) than those who were not physically disciplined [54]. Children’s interpretation of their parents’ use of physical discipline as signs of rejection could be one mediating mechanism behind the negative effects of physical discipline on children’s adjustment. Cultural normativeness has been proposed to influence children’s interpretation of their parents’ use of physical discipline, such that in contexts where physical discipline is widely accepted, children may interpret the practice as a form of care [11, 12]. However, there are also arguments that physical discipline could universally elicit responses of fear, distress, and perception of threat in children, and hence may be linked to detrimental child outcomes regardless of the cultural normativeness of the practice [9]. Further, going beyond children’s immediate interpretations of and reactions to physical discipline, it is unclear how exposure to physical discipline would be related to children’s holistic evaluation of their parents’ parenting (e.g., the extent to which they are caring or controlling) in a society where physical discipline may be widely accepted. It should be noted that children and parents are often discrepant in reporting the parents’ behaviors [55, 56], and children’s interpretation of their parents’ behaviors, including physical discipline, may have more weight in determining subsequent outcomes [57, 58].

The relation between parents’ use of physical discipline and children’s evaluation of their parents’ parenting could differ depending on whether the mother or the father is the parent in question. Siegal and Barclay [59] suggested that children’s perceived legitimacy of parents’ disciplinary methods could differ depending on the gender of the parent, such that physical discipline may be considered more acceptable when used by fathers. Previous literature suggests that fathers are more likely than mothers to take on the role of disciplinarian in Asian cultures [20, 60, 61], which could potentially contribute to children perceiving strict discipline from fathers to be more legitimate. However, inconsistent with previous literature, a study on 10- to 12-year-old children in Singapore found that mothers engaged in more frequent disciplining of their child than did fathers, potentially due to mothers spending more time with their child and thus having more opportunities to become aware of their child’s misbehavior [29]. Being disciplined does not deter the child away from the caregiver, with the same study reporting that children tended to indicate their mother as their preferred caregiver, as they felt that their mother understood them the best and could take better care of them than other caregivers.

## The current study

Our study capitalized on the Growing Up in Singapore Towards healthy Outcomes (GUSTO) study, the most comprehensive birth cohort study in Singapore, where children had repeated parental reports of parenting practices from early to middle childhood. The overarching goal of our study was to examine the normativeness of parental physical discipline in Singapore. We had three specific aims. The first aim of our study was to investigate the prevalence of children exposed to parental physical discipline in any form in Singapore. In addition, we also investigated the prevalence of children being exposed to specific forms of physical discipline (i.e., spanking, slapping, grabbing, and hitting with an object), and examined whether prevalence rates differed by the demographic characteristics of child sex, parent sex, and parent ethnicity. Our second aim was to examine longitudinal changes in the prevalence of parental physical discipline from early to middle childhood. Our third aim was to examine how children’s exposure to parental physical discipline relates to their evaluation of their parents’ parenting, particularly the extent to which they are caring and controlling. We expected that the prevalence of Singaporean children experiencing physical discipline would be high based on previous literature suggesting wide acceptability of the practice. No other specific hypotheses were formulated.

## Method

### Participants

Participants were recruited as part of the GUSTO study, a longitudinal birth cohort study in Singapore. In 2009 and 2010, the GUSTO study recruited 1247 pregnant women in their first trimester at two large public hospitals in Singapore, the National University Hospital and the KK Women’s and Children’s Hospital. The participants recruited were either Singaporean citizens or permanent residents belonging to one of three major ethnicities (i.e., Chinese, Malay, Indian) in Singapore. Details regarding other inclusion and exclusion criteria are available in Soh et al. [62]. After birth, children and their mothers were followed for regular health and metabolic assessments, with fathers also providing data occasionally. The GUSTO study obtained ethical approval from the National Healthcare Group Domain Specific Review Board (D/2009/00021; B/2014/00406; D/2010/00210; B/2014/00414) and the SingHealth Centralised Institutional Review Board (2018/2767/D; 2018/3138/F). Informed consent was provided by all participating mothers and fathers, and participating children have provided assent for all assessments since they reached the age of 6. More than 70% of mothers reported themselves as primary caregivers for their child at assessments of caregiving arrangement when children were age 4.5, 6, and 9 years.

Included in our analyses were children in the “GUSTO Neurodevelopmental Cohort”, a subsample of participants who were invited for regular neuropsychological assessments. Specifically, we included 710 unique children (340 girls, 370 boys) with data on parenting at one or more assessments at ages 4.5, 6, 9, and 11 years. Of the 710 children, 399 had maternal reports on parenting at age 4.5 (*M* = 4.58, *SD* = 0.08), 582 had either maternal or paternal reports of parenting at age 6 (*M* = 5.98, *SD* = 0.12), 414 had maternal reports of parenting and 168 had paternal reports of parenting at age 9 (*M* = 8.91, *SD* = 0.15; *M* = 8.90, *SD* = 0.08; respectively for when maternal and paternal reports were collected), and 393 had maternal reports of parenting at age 11 (*M* = 10.89, *SD* = 0.18). Data on parenting were provided by a total of 701 unique mothers and 202 unique fathers across four assessments of parenting between 2014 and 2022. Of the 710 children included, 403 had maternal reports of parenting and provided reports on their mother’s parenting at age 9, and 160 had paternal reports of parenting and provided reports on their father’s parenting at age 9. Details on exclusion criteria for the present study are reported in the Additional file 1. Table 1 presents the demographic characteristics of the participating parents. Translated questionnaires were provided for participants whose dominant language was not English (i.e., Mandarin, Malay, Tamil).

**Table 1:** Demographic characteristics of participants included in analyses

Demographic Characteristics		Child Sex	
		Girls (*n* = 340)	Boys (*n* = 370)
Maternal Characteristics (701 Total Mothers)	Maternal Age^a^		
	At Child Age 4.5 Assessment, *M* (*SD*) (*n* = 399)	35.84 (5.09)	36.22 (5.10)
	At Child Age 6 Assessment, *M* (*SD*) (*n* = 538)	37.38 (5.33)	37.19 (4.82)
	At Child Age 9 Assessment, *M* (*SD*) (*n* = 414)	40.03 (5.29)	40.07 (4.88)
	At Child Age 11 Assessment, *M* (*SD*) (*n* = 393)	42.07 (5.41)	42.13 (4.96)
	Maternal Ethnicity		
	Chinese %	58	55
	Malay %	26	29
	Indian %	16	15
	Other %	0	0.3
	Maternal Educational Attainment^b^		
	Primary %	5	4
	Secondary %	26	24
	ITE/NITEC %	11	11
	GCE A Levels/Polytechnic/Diploma %	25	24
	University (Bachelors, Masters, PhD) %	33	37
Paternal Characteristics (202 Total Fathers)	Paternal Age^c^		
	At Child Age 6 Assessment, *M* (*SD*) (*n* = 44)	40.44 (5.87)	43.95 (10.02)
	At Child Age 9 Assessment, *M* (*SD*) (*n* = 168)	43.12 (5.66)	44.03 (5.89)
	Paternal Ethnicity^d^		
	Chinese %	61	58
	Malay %	26	27
	Indian %	13	14
	Other %	0	1
	Paternal Educational Attainment^e^		
	Primary %	5	3
	Secondary %	23	19
	ITE/NITEC %	14	18
	GCE A Levels/Polytechnic/Diploma %	19	16
	University (Bachelors, Masters, PhD) %	39	44
Family Characteristics (710 Total Families)	Household Monthly Income (Singapore Dollar)^f^		
	$0-$999 %	3	2
	$1000-$1999 %	15	11
	$2000-$3999 %	29	31
	$4000-$5999 %	26	24
	More than $6000 %	27	33

Parental ethnicity, parental educational attainment, and household income were reported at recruitment. Parental age was calculated using birthdates reported at recruitment and assessment dates

^a^Missing 6 data points at age 4.5 assessment and 2 data points at age 6 assessment. Maternal age at age 9 assessment calculated based on when mothers completed the PSDQ

^b^Missing 8 data points

^c^Missing 1 data point for age 6 assessment and 7 data points for age 9 assessment. Paternal age at age 9 assessment calculated based on when fathers completed the PSDQ

^d^Missing 1 data point

^e^Missing 25 data points

^f^Missing 42 data points

Considering the substantial missingness of data across the four assessments (i.e., at age 4.5, 6, 9, and 11), we conducted preliminary analyses examining whether missingness relates to sociodemographic variables in the sample of children included in our analyses. We found that the number of assessments out of four where children had maternal reports of parenting were not significantly related to sociodemographic characteristics, particularly maternal ethnicity, maternal educational attainment, maternal age, and household income reported at recruitment. Further, children with and without at least one paternal report of parenting did not significantly differ in sociodemographic characteristics, particularly paternal ethnicity, paternal educational attainment, paternal age, and household income reported at recruitment.

### Measures

The Parenting Styles and Dimensions Questionnaire—Short Version (hereafter simply referred to as PSDQ) was used to obtain maternal reports of parenting when children were age 4.5 and 11, and maternal and paternal reports of parenting when children were age 9. The Alabama Parenting Questionnaire (APQ) was used to obtain maternal and paternal reports of parenting when children were age 6. The Parental Bonding Instrument for Children (PBI-C) was used to obtain child reports on their parents’ parenting when they were age 9.

### Parenting styles and dimensions questionnaire—short version

The PSDQ is a 32-item questionnaire that has been used in more than 15 countries and languages to obtain parental reports of how often they engage in practices that are characteristic of authoritative, authoritarian, and permissive parenting styles [63]. The PSDQ was used to obtain parental reports of their own and their spouses’ parenting practices [64]. We used parental reports in the physical coercion dimension, which consisted of four items on the frequency of using physical discipline on a 5-point Likert scale from 1 (‘never’) to 5 (‘always’). The physical coercion dimension included one item on the general use of physical punishment (i.e., ‘I/he/she use(s) physical punishment as a way of disciplining our child’), and three items on the use of the specific physical disciplines of spanking (‘I/he/she spank(s) when our child is disobedient’), slapping (‘I/he/she slap(s) our child when the child misbehaves’), and grabbing (‘I/he/she grab(s) our child when being disobedient’). The PSDQ did not provide a specific time frame (e.g., past year, past month) for participants to base their report on, but the instructions and items were in present tense to have participants report on their behaviors at that time (e.g., ‘This questionnaire is designed to measure how often you exhibit certain behaviors towards this child’).

For maternal reports at the age 4.5, 9, and 11 assessments, Cronbach’s alphas ranged from 0.79 to 0.82 for the full questionnaire and from 0.66 to 0.75 for the physical coercion dimension for self-reports, and from 0.83 to 0.89 for the full questionnaire and from 0.72 to 0.80 for the physical coercion dimension for reports on the spouse. For paternal reports at the age 9 assessment, Cronbach’s alphas were 0.81 for the full questionnaire and 0.74 for the physical coercion dimension for self-reports, and 0.82 for the full questionnaire and 0.79 for the physical coercion dimension for reports on the spouse. For the 188 mothers who completed the PSDQ at all three assessments, their response to each item of the physical coercion dimension was significantly correlated across the assessments, with Pearson’s correlation coefficients ranging from 0.26 to 0.65 (all *p* < 0.001).

### Alabama parenting questionnaire

The APQ is a 42-item questionnaire that has been used in more than 20 countries and languages to obtain parental reports of how often they engage in positive and negative parenting practices [65]. We used parental reports in the corporal punishment subscale, which consisted of three items on the frequency of using physical discipline on a 5-point Likert scale from 1 (‘never’) to 5 (‘always’). The three items in the corporal punishment subscale addressed the three specific physical disciplines of spanking (‘You spank your child with your hand when he/she has done something wrong’), slapping (‘You slap your child when he/she has done something wrong’), and hitting with an object (‘You hit your child with a belt, switch (cane), or other object when he/she has done something wrong’). Similar to the PSDQ, the APQ did not provide a specific time frame for participants to base their report on, but the instructions and items were in present tense to assess participants’ behaviors at that time. Cronbach’s alphas were 0.73 for the full questionnaire and 0.56 for the corporal punishment subscale for maternal reports, and 0.73 for the full questionnaire and 0.63 for the corporal punishment subscale for paternal reports.

### Parental bonding instrument for children

The PBI-C [66], a modification of the Parental Bonding Instrument [67], is a 25-item questionnaire used to obtain children’s reports on their parents’ parenting. Based on the past year, children rated each of their parents on statements pertaining to their level of care (12 items) and overprotection (13 items) on a 4-point Likert scale from 0 (‘not true’) to 3 (‘very true’). However, Cronbach’s alphas were a low 0.50 and 0.61 respectively for maternal and paternal overprotection, with the seven non-reverse coded items (e.g., “Does not want me to grow up”) and six reverse-coded items (e.g., “Lets me do the things I like to do”) tending to show patterns of negative correlations with each other. As such, we split the non-reverse-coded items and reverse-coded items, which we will refer to as denial of psychological autonomy and denial of behavioral freedom respectively (i.e., dimension names adapted from Murphy et al. [68]). Cronbach’s alphas were 0.64 and 0.63 respectively for maternal and paternal denial of psychological autonomy, and 0.74 and 0.71 respectively for maternal and paternal denial of behavioral freedom. Cronbach’s alphas were an adequate 0.73 and 0.76 respectively for maternal and paternal care, despite the subscale also being comprised of a mix of non-reverse-coded items (e.g., “Speaks to me with a warm and friendly voice”) and reverse-coded items (e.g., “Makes me feel I’m not wanted”).

### Statistical analyses

To address the first aim of our study, we examined the prevalence of physical discipline of children at four different ages using parental self-reports of physical discipline in the PSDQ at the age 4.5, 9, and 11 assessments, and the APQ at the age 6 assessment (see Participants section for the number of parental reports at each assessment). For each age of assessment, we computed the percentage of children by parents’ self-reported frequency of using each physical discipline (e.g., spank, slap, hit with object). The prevalence of each physical discipline was computed as the percentage of children whose parents reported using the discipline regardless of their frequency of use (i.e., reported any frequency aside from ‘never’). We further computed the prevalence of children being physically disciplined in any form as the percentage of children whose parents endorsed at least one physical discipline regardless of their frequency of use.

Additionally, for each age of assessment, we computed the percentage of children by the number of specific physical disciplines out of three that their parents reported using. We also used data from 164 children with maternal self-reports of physical discipline in all assessments at ages 4.5, 6, 9, and 11 to compute the number of assessments out of four that each child was physically disciplined in any form by their mothers.

We also examined whether the percentage of children exposed to at least one form of physical discipline, as self-reported by parents, differed by participant demographics. First, we used chi-square tests to examine whether prevalence rates differed by child sex at each age of assessment. Second, we conducted McNemar’s tests to investigate whether prevalence rates differed by parent sex, using data from 162 children who had both maternal and paternal self-reports of physical discipline in the PSDQ at the age 9 assessment. Third, we used chi-square tests to examine whether prevalence rates of physical discipline by mothers differed by maternal ethnicity at each age of assessment.

To address the second aim of our study, we investigated the change in the prevalence of physical discipline from early to middle childhood in 611 children with maternal reports on their own and the fathers’ use of physical discipline in the PSDQ, and data on age at the time of assessment, in at least one assessment at age 4.5, 9, and 11 years. A generalized linear mixed model with binomial error structure, logit link function, and maximum likelihood estimation was conducted to examine whether age at each assessment predicts children’s exposure to physical discipline as a binary variable (i.e., 0 = never used, 1 = used once in a while or more frequently). We entered children’s age at each assessment, parent sex (mother or father), physical coercion dimension item (general use of physical punishment, spank, slap, or grab), and the two-way interaction terms of children’s age with parent sex and physical coercion dimension item as fixed effects in the model. Participant ID was included as a random effect variable to account for the repeated measures nature of the data.

To address the third aim of our study, we examined the relation between parents’ self-reported frequency of using physical discipline in the PSDQ and children’s reports on their parents’ parenting in the PBI-C at age 9. Analyses examining the relation between children’s exposure to maternal physical discipline and evaluation of their mother’s parenting were conducted with a subset of 403 children who evaluated their mother in the PBI-C and whose mother had completed the PSDQ at the age 9 assessment. Analyses examining the relation between children’s exposure to paternal physical discipline and evaluation of their father’s parenting were conducted with a subset of 160 children who evaluated their father in the PBI-C and whose father had completed the PSDQ at the age 9 assessment. For the PSDQ, the physical coercion dimension score, or the average of parents’ responses (i.e., 1 to 5) to the four items on physical discipline, were used for analyses. For the PBI-C, the sum of children’s responses (i.e., 0 to 3) to items on each parent’s care, denial of psychological autonomy, and denial of behavioral freedom were used for analyses. Simple linear regression analyses were conducted to test whether the physical coercion dimension score in the PSDQ predicts scores in the PBI-C, separately for each dimension of parenting in the PBI-C. Holm-Bonferroni adjusted *p* values were calculated to account for three tests for each dimension in the PBI-C.

## Results

### Reliability of parental self-reports

Considering that self-reports of physical discipline are subject to socially desirable responding tendencies [69], we examined the consistency between parental self- and spouse-reports in 162 children who had both maternal and paternal reports on their own and their spouses’ use of physical discipline in the PSDQ at the age 9 assessment. Details are reported in the Additional file 1. We did not find evidence of severe non-disclosure in self-reports. For example, there was only one out of 162 children (0.62%) whose mother or father reported never using physical punishment, while their spouse reported that they used physical punishment often (i.e., collapsed ‘about half of the time’, ‘very often’, and ‘always’). More minor discrepancies were common, leading to slight to moderate agreement between parental self- and spouse-reports (κ ranging from 0.19 to 0.42 across PSDQ items). Such minor discrepancies between self- and spouse- reports were comprised of a mix of cases where physical discipline was underreported in self-reports and cases where physical discipline was overreported in self-reports.

### Prevalence of physical discipline of children by their parents

Table 2 presents, for each age of assessment, the percentage of children by their parents’ self-reported frequency of using each physical discipline, the prevalence of each physical discipline, and the prevalence of any form of physical discipline. Across the four assessments, the percentage of children whose parents reported using at least one physical discipline ranged from 81 to 94%. At each assessment, parents who used each physical discipline tended to report using them at lower frequencies such as ‘once in a while’ or ‘sometimes’, with a minority of parents reporting using them at high frequencies such as ‘often’ or ‘always’. Table 3 presents, for each age of assessment, the percentage of children in relation to the number of specific physical disciplines out of three that their parents reported using. Across the four assessments, up to 32% of parents endorsed using all three forms of physical discipline.

**Table 2:** Prevalence of physical discipline of children at age 4.5, 6, 9, and 11

Age of Assessment (Questionnaire)^a^	Parent/Physical Discipline in Subscale	Frequency Reported by Parents^b^					
		1. Never	2. Once in a While/Almost Never	3. About Half of the Time/Sometimes	4. Very Often/Often	5. Always	Prevalence^c^
Age 4.5 (PSDQ)	Mother (*n* = 399)						
	Physical Punishment, General %	16	56	16	10	3	84
	Spank %	19	44	21	11	5	81
	Slap %	57	33	6	3	2	43
	Grab %	42	35	12	9	2	58
	**Any Form of Physical Discipline %**^d^	—	—	—	—	—	**94**
Age 6 (APQ)	Mother (*n* = 538)						
	Spank %	10	17	60	10	3	90
	Slap %	45	29	25	1	0	55
	Hit With Object %	53	16	26	4	1	46
	**Any Form of Physical Discipline %**^d^	—	—	—	—	—	**93**
	Father (*n* = 44)						
	Spank %	14	20	55	9	2	86
	Slap %	50	23	25	2	0	50
	Hit With Object %	64	7	25	5	0	36
	**Any Form of Physical Discipline %**^d^	—	—	—	—	—	**86**
Age 9 (PSDQ)	Mother (*n* = 414)						
	Physical Punishment, General %	23	53	14	7	4	77
	Spank %	27	47	13	8	5	73
	Slap %	61	31	5	2	1	39
	Grab %	50	32	11	6	2	50
	**Any Form of Physical Discipline %**^d^	—	—	—	—	—	**88**
	Father (*n* = 168)						
	Physical Punishment, General %	24	48	15	8	5	76
	Spank %	27	41	18	11	2	73
	Slap %	68	22	8	1	1	32
	Grab %	47	32	15	5	1	53
	**Any Form of Physical Discipline %**^d^	—	—	—	—	—	**87**
Age 11 (PSDQ)	Mother (*n* = 393)						
	Physical Punishment, General %	32	53	9	5	1	68
	Spank %	33	50	8	7	2	67
	Slap %	70	26	2	1	1	30
	Grab %	58	32	6	3	1	42
	**Any Form of Physical Discipline %**^d^	—	—	—	—	—	**81**

^a^Data from the physical coercion dimension of the PSDQ at the age 4.5, 9, and 11 assessments, and from the corporal punishment subscale of the APQ at the age 6 assessment

^b^Percentage of children by parents’ self-reported frequency of using the physical discipline. Labels for response scales 2 to 4 differed by questionnaire. Labels for the PSDQ are provided before the forward slash, and labels for the APQ are provided after the forward slash

^c^Percentage of children whose parents endorsed using the physical discipline, regardless of frequency of use

^d^In bold font are the percentages of children whose parents, regardless of frequency, endorsed using at least one physical discipline

**Table 3:** Number of specific physical disciplines endorsed by parents at each assessment

Age of Assessment (Questionnaire)/Parent	Number of Specific Physical Disciplines Endorsed			
	None %^a^	One %^a^	Two %^a^	Three %^a^
Age 4.5 (PSDQ)^b^				
Mother (*n* = 399)	10	28	31	31
Age 6 (APQ)^c^				
Mother (*n* = 538)	7	26	36	32
Father (*n* = 44)	14	25	36	25
Age 9 (PSDQ)^b^				
Mother (*n* = 414)	17	30	27	26
Father (*n* = 168)	20	27	30	23
Age 11 (PSDQ)^b^				
Mother (*n* = 393)	26	30	25	19

^a^Percentage of children in relation to the number of specific physical disciplines out of three that their parents reported using, regardless of frequency of use

^b^In the PSDQ at the age 4.5, 9, and 11 assessments, parents were asked regarding their use of spanking, slapping, and grabbing

^c^In the APQ at the age 6 assessment, parents were asked regarding their use of spanking, slapping, and hitting with an object

Of the 164 children with maternal self-reports of physical discipline for all assessments at ages 4.5, 6, 9, and 11, there was only one child (0.6%) whose mother reported not using any form of physical discipline (i.e., selected ‘never’ for all physical discipline items) across all four assessments. The remaining 163 children were exposed to at least one physical discipline by their mothers in at least one assessment. Specifically, 121 children (74%) were physically disciplined by their mother at all four assessments, 29 children (18%) at three assessments, 10 children (6%) at two assessments, and 3 children (2%) at one assessment only.

The prevalence of experiencing at least one form of physical discipline did not differ by child sex at all four ages of assessment (i.e., maternal self-reports at all assessments, and paternal self-report at age 9), with prevalence rates ranging from 82 to 96% for girls and from 80 to 94% for boys (χ^2^ ≤ 2.23, *p* ≥ 0.14). Further, prevalence rates did not significantly differ by parent sex, with 88% of children being physically disciplined by their mothers and 86% being physically disciplined by their fathers among the 162 children who had maternal and paternal self-reports of physical discipline at the age 9 assessment (χ^2^ = 0.29, *p* = 0.59). Finally, the prevalence of children experiencing at least one form of physical discipline did not significantly differ by maternal ethnicity at the age 4.5, 6, and 11 assessments, with prevalence rates ranging from 82 to 94% for Chinese, 77 to 94% for Malay, and 86 to 97% for Indian mothers (χ^2^ ≤ 1.95, *p* ≥ 0.38). However, the prevalence of any form of physical discipline significantly differed by maternal ethnicity at the age 9 assessment, with this prevalence being highest among Malay mothers (i.e., 85% for Chinese, 94% for Malay, and 91% for Indian mothers; χ^2^ = 7.22, *p* = 0.03). There were subtle differences in prevalence rates according to parent sex and maternal ethnicity, but not child sex, when specific forms of physical discipline were examined (see Additional file 1). Notably, slapping tended to be most prevalent among Indian mothers at the age 4.5, 6, and 9 assessments, whereas hitting with an object tended to be most prevalent among Chinese mothers at the age 6 assessment.

### Change in prevalence of physical discipline of children from early to middle childhood

Table 4 shows the percentage of children who were physically disciplined by their parents at any frequency when they were ages 4.5, 9, and 11. The generalized linear mixed model conducted suggested a significant effect of children’s age, such that children were less likely to be physically disciplined as they became older, (B = -0.14, *SE* = 0.01, χ^2^(1) = 153.43, *p* < 0.001, OR = 0.87, 95% CI [0.85, 0.89]). The effects of the interaction of children’s age with parent sex and physical coercion dimension item did not achieve significance (*p*s ≥ 0.21), suggesting that the decrease in the prevalence of physical discipline with age did not differ by parent sex or by the four items of the physical coercion dimension. We additionally conducted a linear mixed model with restricted maximum likelihood estimation entering the same fixed effects as in the model described above, but with children’s frequency of exposure to physical discipline (i.e., on a Likert scale from 1 = ‘never’ to 5 = ‘always’) as the dependent variable. This model similarly yielded a significant effect of age, such that children’s frequency of exposure to physical discipline decreased with age (B = − 0.05, *SE* = 0.003, *F*(1, 9527.78) = 222.53, *p* < 0.001). The interaction between age and parent was nonsignificant (*F*(1, 8982.68) = 1.36, *p* = 0.24), but the interaction between age and physical coercion dimension item was significant (*F*(3, 8982.68) = 5.38, *p* = 0.001), such that the decline in frequency with age was largest for spanking and smallest for slapping. It should be noted that children’s age remained a significant predictor of children’s exposure to parental physical discipline even after controlling for children’s cognitive skills at age 4.5 years (see Additional file 1), which we considered as potentially relevant based on previous literature speculating that parents may rely less on physical discipline over time as children develop cognitive maturity. [43, 44]

**Table 4:** Percentage of children physically disciplined at age 4.5 (n = 399), 9 (n = 414), and 11 (n = 393)

Physical Coercion Dimension Item	Mothers (Self-Report)			Fathers (As Reported by Mothers)		
	Age 4.5	Age 9	Age 11	Age 4.5	Age 9	Age 11
Physical Punishment, General %^a^	84	77	68	71	64	54
Spank %^a^	81	73	67	71	59	52
Slap %^a^	43	39	30	28	22	18
Grab %^a^	58	50	42	51	41	30
**Any Form of Physical Discipline %**^**b**^	**94**	**88**	**81**	**83**	**78**	**67**

^a^Percentage of children whose parents used the physical discipline, regardless of frequency of use, according to maternal reports

^b^In bold font are the percentages of children whose parents, regardless of frequency, used at least one physical discipline according to maternal reports

### Relation between children’s exposure to physical discipline and evaluation of parenting

The regression analyses conducted revealed that mothers’ self-reported frequency of using physical discipline was not significantly related to how children rated their mothers’ parenting, particularly their care (*R*^2^ = 0.00, *F*(1, 401) = 1.83, B = − 0.55, *SE* = 0.41, nominal *p* = 0.18, adjusted *p* = 0.53), denial of psychological autonomy (*R*^2^ = 0.00, *F*(1, 401) = 0.19, B = 0.14, *SE* = 0.32, nominal and adjusted *p* = 0.66), and denial of behavioral freedom (*R*^2^ = 0.00, *F*(1, 401) = 1.72, B = 0.38, *SE* = 0.29, nominal *p* = 0.19, adjusted *p* = 0.53). In contrast, more frequent self-reported use of physical discipline by fathers was significantly associated with children reporting lower levels of care (*R*^2^ = 0.04, *F*(1, 158) = 6.92, B = − 1.74, *SE* = 0.66, nominal *p* = 0.01, adjusted *p* = 0.03) and higher levels of denial of psychological autonomy (*R*^2^ = 0.03, *F*(1, 158) = 5.59, B = 1.05, *SE* = 0.45, nominal *p* = 0.02, adjusted *p* = 0.04) by fathers. Fathers’ self-reported use of physical discipline was not significantly related to how children rated their fathers’ denial of behavioral freedom (*R*^2^ = 0.00, *F*(1, 158) = 0.40, B = 0.26, *SE* = 0.41, nominal and adjusted *p* = 0.53). While the analyses above examined concurrent relations between exposure to physical discipline and evaluation of parenting at age 9, supplementary analyses suggested that children’s exposure to maternal physical discipline at age 4.5 and 6 were also not significantly associated with how they evaluated their mothers’ parenting at age 9 (see Additional file 1).

Our main analyses above examined the extent to which parents’ self-reported frequency of using physical discipline was associated with children’s evaluation of their parents as caring and controlling. Considering that child- and parent-reports of parenting can often be discrepant [55], we conducted supplementary analyses examining whether parents’ self-reported frequency of using physical discipline was associated with their self-reported frequency of engaging in parenting behaviors reflecting care (i.e., warmth) and control (i.e., autonomy granting) towards the child. Details are reported in the Additional file 1. Pearson’s correlation analyses using parental self-reports in the PSDQ at the age 9 assessment suggested that mothers’ use of physical discipline was significantly and negatively associated with their warmth, but not significantly related to their autonomy granting. Fathers’ use of physical discipline was significantly and negatively associated with both their warmth and autonomy granting.

## Discussion

To date, there have been no studies establishing the prevalence of physical discipline among children in Singapore, although previous studies have suggested that physical discipline of children is widely accepted [30, 31]. The present study capitalized on the GUSTO study, a comprehensive birth cohort in Singapore. We found that most children had experienced at least one instance of physical discipline by their parents, with the prevalence of physical discipline exceeding 80% across the four assessments when children were age 4.5, 6, 9, and 11 years. Our findings suggest that physical discipline could be a common form of discipline experienced by children in Singapore and may constitute a normative practice of parents as assessed until 2022.

The functional meaning conveyed by specific parenting practices differs according to the cultural context in which it occurs [15]. A recent review of studies on parenting in Singapore concluded that authoritarian parenting, characterized by strictness and focus on obedience, is not necessarily detrimental to developmental outcomes for Singaporean children [23]. The authors postulated that strict and controlling parenting practices may not be regarded as a form of hostility but could rather be perceived as a sign of parental concern with the child’s best interests in mind, an idea that has been discussed in other literature on parenting in Singapore [26–28, 70] and described more extensively in literature on Chinese parenting [19–21]. Physical discipline could also be one component of strict parenting that is practiced as an act of care and a part of responsible child-rearing [36, 71, 72]. The Chinese proverb “Hitting is concern, scolding is love” which exemplifies this idea has been alluded to in previous studies finding a high prevalence of physical discipline in mainland China (i.e., around 45% to 80% for 4- to 9-year-olds [40]; 58% for children who were 9 years old on average [73]) and Hong Kong (i.e., 74% for children who were 9 years old on average [74]), as well as an article discussing the widespread acceptance of caning in Singapore. [28]

Children in Singapore are provided with formal protection against abuse. For example, the Children and Young Persons Act states that one will be guilty of offense if they ill-treat a child, which includes any act that causes or is likely to cause “unnecessary physical pain, suffering, or injury” [75]. However, Ngiam and Tung [28] state that there is no explicit definition of what constitutes physical abuse in Singapore and suggests that this ambivalence could be rooted in the widespread acceptance of physical discipline in the local context. They also postulate that the acceptability of caning children in Singapore could partially be influenced by the local use of caning as a judicial penalty, a vestige of the British colonial judicial system. However, not all forms of physical discipline are invariably accepted in Singapore, with a previous study showing that the majority of Singaporean adults considered caning to be acceptable only under certain conditions, such as when it is done infrequently, only the limbs and buttocks are affected, and no permanent marks or injuries are left [30].

The use of physical discipline is certainly not limited to the Chinese majority in Singapore, with our study finding that high prevalence rates were also shared by the Malay and Indian minorities. While the concept of filial piety (*xiao*), which includes respect and courtesy to parents, is rooted in Chinese culture, similar values may be shared by the Malay culture (*ketaatan kepada ibu bapa*) and Indian culture (*seva*) [76]. Prevalence aside, the specific physical discipline parents choose to use could differ by ethnicity, considering our preliminary findings that the use of slapping was most prevalent among Indian mothers across multiple age assessments, whereas hitting with an object was most prevalent among Chinese mothers at the age 6 assessment. The underlying mechanism for ethnic differences in the form of physical discipline used is difficult to speculate, and thus an avenue for future research, considering that previous studies comparing physical discipline experiences across demographic groups (i.e., country, ethnicity, race) have typically focused on whether physical discipline occurs and how often, rather that the quality of its occurrence [38, 39, 77].

Our study was unable to capture a fine-grained picture of children’s physical discipline experiences. While many Singaporean children may have been physically disciplined by their parents, there can be wide variability in the delivery of physical discipline, including its frequency, severity, form, and impulsivity, as well as whether it is preceded by milder disciplinary techniques and accompanied by verbal reasoning. Circumstances that elicit parental physical discipline and the motivations behind parents' use of the practice could also differ across households, considering previous literature suggesting that parents' disciplinary choices (e.g., use of harsh versus non-harsh discipline in response to child anger) could differ depending on parents’ socialization goals (e.g., emphasis on teaching children to control negative emotions) [78]. Our study provides preliminary evidence of variability in parental reports of children’s physical discipline experiences. We found that most parents indicated that they used physical discipline ‘once in a while’ or ‘sometimes’, while a minority of parents endorsed physical discipline at high frequencies such as ‘often’ or ‘always’. As for children’s exposure to specific physical disciplines, we found that spanking was the most common form of physical discipline at each assessment, whereas more severe physical disciplines such as slapping and hitting with an object were used by a smaller proportion of parents. However, the PSDQ and APQ were developed in the United States, limiting their capability to capture culture-specific experiences of parenting. Neither questionnaire has an item specific to caning, which may be a more commonly experienced form of physical discipline in Singapore [28, 79], compared to spanking, the most common physical discipline in the United States [4]. Finally, some parents reported using all three specific physical disciplines addressed in the PSDQ or APQ, whereas other parents reported using only one of the physical disciplines addressed. Future research on the nature and implications of physical discipline in Singapore should employ measures that can better capture the variability of children’s physical discipline experiences. Further, child-reports of parental physical discipline could be collected in addition to parental self-reports. Combining information from multiple sources could provide more comprehensive information on children’s physical discipline experiences, considering that all informants’ reports are imperfect [80], and self-reports of parenting can be affected by social desirability biases [69]. Moreover, children’s perception of their physical discipline experiences could carry more weight in determining subsequent developmental outcomes [11, 57].

Previous research found that boys are more subject to physical discipline than girls, which could be due to factors such as gender expectations that boys require harsher discipline, or higher expectations for boys to be leaders within their household and the society in the future [35–37]. However, we found no significant differences by child sex in the prevalence of experiencing at least one instance of physical discipline. Filial piety, obedience, and academic success are strongly emphasized in Singapore [51], and high expectations for these characteristics could potentially contribute to parents’ use of strict discipline towards their children regardless of their sex. Previous studies also commonly showed that mothers are more likely than fathers to physically discipline their child, potentially due to mothers spending more time with their child [38–40]. Mothers are typically primary caregivers to children in Singapore [29], but the rates at which children in our sample experienced at least one instance of physical discipline by their mothers and fathers at age 9 were similar. The high prevalence of physical discipline by fathers despite their comparatively lower involvement in caregiving could be consistent with previous literature suggesting that fathers are more likely than mothers to take on the role of strict disciplinarian in Asian cultures [20, 60, 61]. However, there remains a possibility that the roles of mothers and fathers in caregiving could differ by children’s age, with a previous study on older Singaporean children (i.e., 10- to 12-year-olds) finding that mothers engaged in more frequent disciplining of their child than did fathers [29]. Finally, the caregiving culture in Singapore is rather unique in general. Most married couples are dual-career couples [81] and tend to receive caregiving support from grandparents, paid workers, or both (e.g., 70% of households with preschool children) [82]. The prevalence of parental physical discipline in Singapore is intriguing if considering previous suggestions that time spent with children could be associated with greater use of physical discipline.

Previous studies, mostly conducted in Western countries, suggest that the prevalence of parental physical discipline tends to be high in early childhood and declines thereafter, potentially as children develop the sophisticated cognitive skills to reflect on and control their misbehavior, and mutual decision making becomes more prioritized in the parent–child relation [35, 39, 44–47, 83]. In our study, we found that the prevalence of physical discipline declined from age 4.5 to 11 years, rather than rising with potential increases in academic demands as Singaporean children navigate formal schooling and prepare for a high-stakes national examination taken at age 12. Thus, our findings are consistent with previous studies that suggest that the use of physical discipline decreases from early to middle childhood. However, it should be noted that there could be more subtle differences between cultural contexts in changes in exposure to physical discipline over childhood. For example, one study in mainland China found that the prevalence of physical discipline decreased by late childhood in consistency with previous studies on Western countries [40]. However, rather than peaking in the preschool years, the prevalence of physical discipline peaked at the start of formal schooling, potentially due to Chinese parents’ growing academic and behavioral expectations for their child during this time. A limitation with our study is that it was not adequately designed to determine fine-grained peaks and valleys in the prevalence of physical discipline over childhood, as the PSDQ was administered at unequal intervals, with at least a few years of interval between each assessment. Further, future research could elucidate whether within-person variance in cognitive skills and parent–child relation is associated with that of exposure to physical discipline.

Physical discipline could be interpreted by children as parental hostility or rejection, particularly in cultural contexts where physical discipline is non-normative [11, 12]. A recent study found that young adults who experienced physical discipline during their childhood were less likely than those who had no such experience to report positive evaluations of the parenting (e.g., loving, good as other parents) they received [54]. The study also found that parent gender was influential, such that an interaction effect emerged between participants’ exposure to physical discipline (i.e., from neither parent, by father, by mother, by both parents) and the gender of the parent being evaluated. Specifically, when physically disciplined by either their mother or father, young adults rated the parenting of the parent that used physical discipline more negatively than that of the parent that did not use physical discipline, but the magnitude of this difference was larger when they were physically disciplined by their father compared to when they were physically disciplined by their mother. Our study found that the more frequently fathers used physical discipline, the more likely children were to report lower levels of care and higher levels of denial of psychological autonomy by their fathers. This was paralleled by fathers being less likely to report engaging in behaviors reflecting warmth and autonomy granting the more they reported using physical discipline. In contrast, the frequency by which children were physically disciplined by their mothers was not significantly related to their reports of their mother’s care and denial of psychological autonomy and behavioral freedom.

The impact of maternal physical discipline on children could be offset by the comparatively larger quantity of mother–child interactions that occur in a child’s daily life. In Singapore, mothers tend to be main caregivers to children and are more engaged than fathers in a wide variety of parenting practices that reflect involvement in their child’s life, warmth and acceptance towards the child, and guidance of the child [29]. Previous research also suggests that maternal authoritarian parenting seems to have less negative connotations, and less negative implications for developmental outcomes, in comparison to paternal authoritarian parenting in the Singaporean context [23, 26]. Nonetheless, we found that being exposed to physical discipline does not translate to children reporting their parent to be caring, although strict parenting is often described in previous literature on Chinese parenting and parenting in Singapore as a form of care and concern for the child [19–21, 26–28]. It should be noted that rather than attributing our findings to potential differences in the impacts of maternal and paternal physical discipline, it is also possible to interpret the results as reflecting that mothers and children may not show agreement in reporting of parenting behaviors, whereas fathers and children tend to show partial agreement in doing so. However, such an interpretation is difficult to explain, particularly in consideration that a meta-analysis found that children tend to show modest agreement with parents regarding their parenting behavior, regardless of whether it is the mother or father in question [55]. Further, it should be noted that our findings are correlational and cannot yield causal inferences. For instance, it is possible that negative father-child relationships could result in more frequent physical discipline by fathers, rather than the other way around [53].

This study suggests that the majority of children in Singapore experience at least one instance of parental physical discipline. What should follow is a careful examination of the implications of physical discipline, including the nature of its delivery, for child development given the Singaporean sociocultural context. The outcomes of a given discipline could depend on how children interpret the messages conveyed by the practice [11]. One study found that 10- to 12-year-old Singaporean children, on average, indicated neutrality towards physical discipline, evaluating the practice to be neither fair nor unfair and neither effective nor ineffective [84]. However, previous studies suggest that exposure to physical discipline can be linked to detrimental child outcomes across cultural contexts, even where the practice is considered normative and hence potentially less likely to be interpreted as hostile [9, 12]. Further, two studies provide preliminary suggestions that physical discipline could be associated with child aggression in Singapore. However, the findings need to be taken with caution as one study involved a clinical sample [85] and the other assessed parents’ endorsement of physical discipline in hypothetical scenarios rather than their actual use [79]. It should be noted that physical discipline is not the predominantly used disciplinary method in Singapore, with a previous study reporting that Singaporean parents used verbal reasoning most frequently, and perceived the practice to be more effective than physical discipline in addressing their child’s misbehavior [29, 84]. Considering that parents engage in an amalgam of parenting practices, future research should comprehensively examine how physical discipline, in conjunction with other disciplinary methods (i.e., nonviolent discipline and psychological aggression), could impact child development in Singapore. Future work may also wish to consider whether the impact of physical discipline is moderated by the use of “repair” behaviors, such as expressions of warmth and acceptance following physical discipline.

Parents deserve extensive and compelling research on the potential outcomes of their disciplinary decisions, prior to being made recommendations about how their parental ‘toolbox’ techniques should be modified [7]. It will also be essential to examine the motivation behind parents’ use of physical discipline in Singapore, children’s and parents’ perception of the disciplinary practice, and which parents are particularly prone to relying on more frequent and severe forms of physical discipline. Such culture-specific empirical information will form the basis of constructive dialogue on whether and how the use of alternative disciplinary methods should be promoted to support parents in Singapore to foster healthy development in their children. We hope that our findings, in establishing that physical discipline could be a common childhood experience in Singapore, serve as an important knowledge base for future empirical investigations on the nature and implications of physical discipline of children in Singapore.

## Supplementary Information

**Additional file 1: Table S1**. Contingency Table for Frequency at Which Children Were Physically Punished by Their Mothers as Reported by Their Mothers and Fathers. **Table S2**. Contingency Table for Frequency at Which Children Were Physically Punished by Their Fathers as Reported by Their Fathers and Mothers. **Table S3**. Contingency Table for Frequency at Which Children Were Spanked by Their Mothers as Reported by Their Mothers and Fathers. **Table S4**. Contingency Table for Frequency at Which Children Were Spanked by Their Fathers as Reported by Their Fathers and Mothers. **Table S5**. Contingency Table for Frequency at Which Children Were Slapped by Their Mothers as Reported by Their Mothers and Fathers. **Table S6**. Contingency Table for Frequency at Which Children Were Slapped by Their Fathers as Reported by Their Fathers and Mothers. **Table S7**. Contingency Table for Frequency at Which Children Were Grabbed by Their Mothers as Reported by Their Mothers and Fathers. **Table S8**. Contingency Table for Frequency at Which Children Were Grabbed by Their Fathers as Reported by Their Fathers and Mothers. **Table S9**. Prevalence of Physical Discipline of Children by Their Parents at Age 4.5, 6, 9, and 11 According to Child Sex. **Table S10**. Prevalence of Physical Discipline of Children by Their Mothers and Fathers at Age 9. **Table S11**. Prevalence of Physical Discipline of Children by Their Mothers at Age 4.5, 6, 9, and 11 by Maternal Ethnicity.

## Data Availability

Data are from the Growing Up in Singapore Towards healthy Outcomes study and are not publicly available due to confidentiality requirements imposed by the local ethics review board. Data can be requested by contacting GUSTO’s data team manager, Mukkesh Kumar, at mukkesh_kumar@sics.a-star.edu.sg. The GUSTO executive committee reviews the data access request and approves the distribution of data.
