# Supplementary material for: Physical discipline as a normative childhood experience in Singapore
Source: Child Adolesc Psychiatry Ment Health. 2023 Jun 29;17:81. doi: 10.1186/s13034-023-00632-9 (PMC10311744; doi:10.1186/s13034-023-00632-9)
Supplement: Supplementary file 1 — Additional file 1: Table S1. Contingency Table for Frequency at Which Children Were Physically Punished by Their Mothers as Reported by Their Mothers and Fathers. Table S2. Contingency Table for Frequency at Which Children Were Physically Punished by Their Fathers as Reported by Their Fathers and Mothers. Table S3. Contingency Table for Frequency at Which Children Were Spanked by Their Mothers as Reported by Their Mothers and Fathers. Table S4. Contingency Table for Frequency at Which Children Were Spanked by Their Fathers as Reported by Their Fathers and Mothers. Table S5. Contingency Table for Frequency at Which Children Were Slapped by Their Mothers as Reported by Their Mothers and Fathers. Table S6. Contingency Table for Frequency at Which Children Were Slapped by Their Fathers as Reported by Their Fathers and Mothers. Table S7. Contingency Table for Frequency at Which Children Were Grabbed by Their Mothers as Reported by Their Mothers and Fathers. Table S8. Contingency Table for Frequency at Which Children Were Grabbed by Their Fathers as Reported by Their Fathers and Mothers. Table S9. Prevalence of Physical Discipline of Children by Their Parents at Age 4.5, 6, 9, and 11 According to Child Sex. Table S10. Prevalence of Physical Discipline of Children by Their Mothers and Fathers at Age 9. Table S11. Prevalence of Physical Discipline of Children by Their Mothers at Age 4.5, 6, 9, and 11 by Maternal Ethnicity. [file 13034_2023_632_MOESM1_ESM.docx]

**Physical Discipline as a Normative Childhood Experience in Singapore**

**Supplementary Online Material**

**Exclusion Criteria**

At each assessment of parenting at ages 4.5, 6, 9, and 11 years, participants were excluded from analyses according to the following criteria: (a) Not all items in the questionnaire were completed, (b) the questionnaire was completed by the parent whom the questionnaire was not intended for (i.e., father responding to questionnaire phrased for mother), (c) the questionnaire was completed by a non-parent caregiver (i.e., sister), or (d) there were no records of who completed the questionnaire. Further, there were three sets of twins who participated in at least one of the four assessments of parenting at ages 4.5, 6, 9, and 11 years. Parents were asked to complete the questionnaire at a given assessment twice based on their experiences with each twin. We decided to exclude one twin in each set of twins across the four assessments. For two sets of twins, one twin was randomly selected to be excluded. For one set of twins, one twin was selected to be excluded due to missing data at one assessment of parenting in which the other twin had complete data.

At the age 4.5 assessment, when the Parenting Styles and Dimensions Questionnaire – Short Version (PSDQ) was used to obtain maternal reports of parenting, data of 30 participants were excluded due to incomplete questionnaire data (*n* = 14; one of whom also had questionnaire data that was completed by the father), the questionnaire data having no record of who completed it (*n* = 2), the questionnaire being completed by the father (*n* = 12), and the data belonging to a twin that was selected to be excluded (*n* = 2). Participants were included in cases where the PSDQ was completed as a collaborative effort between the mother and the father (*n* = 5).

At the age 6 assessment, when the Alabama Parenting Questionnaire (APQ) was used to obtain maternal and paternal reports of parenting, data of six participants were excluded due to incomplete questionnaire data (*n* = 1), the questionnaire data having no record of who completed it (*n* = 1), the questionnaire being completed by a non-parent caregiver (*n* = 2), and the data belonging to a twin that was selected to be excluded (*n* = 2).

At the age 9 assessment, for the PSDQ used to obtain maternal reports of parenting, data of two participants were excluded due to the questionnaire being completed by the father (*n* = 1), and the data belonging to a twin that was selected to be excluded (*n* = 1). At the age 9 assessment, for the PSDQ used to obtain paternal reports of parenting, four participants were excluded due to incomplete questionnaire data.

At the age 11 assessment, when the PSDQ was used to obtain maternal reports of parenting, data of 16 participants were excluded due to the questionnaire being completed by the father (*n* = 10), the questionnaire being completed by a non-parent caregiver (*n* = 1), incomplete questionnaire data (*n* = 3), and the data belonging to a twin that was selected to be excluded (*n* = 2).

Finally, for analyses on the relation between fathers’ frequency of physical discipline use and children’s evaluation of their fathers’ parenting at the age 9 assessment, two data points were excluded due to incomplete questionnaire data (i.e., missing responses for ratings of father’s parenting) in the Parental Bonding Instrument for Children (PBI-C).

**Informant Consistency in Children’s Exposure to Specific Physical Disciplines**

We examined informant consistency in the frequency at which children were physically disciplined by their parents by comparing parents’ self- and spouse-reports via contingency tables. We included 162 children who had both maternal and paternal reports on their own and their spouses’ use of physical discipline in the PSDQ at the age 9 assessment. For the purpose of this analysis, the responses on the original 5-point Likert scale were categorized into three frequencies: (1) never, (2) once in a while, and (3) often (i.e., we collapsed ‘about half of the time’, ‘very often’, and ‘always’ into 'often'). Contingency tables on patterns of consistency in parental self- and spouse-reports of the frequency at which children were physically disciplined by mothers and fathers are presented respectively in Tables S1 and S2 for the PSDQ item on general use of physical punishment, Tables S3 and S4 for the item on spanking, Tables S5 and S6 for the item on slapping, and Tables S7 and S8 for the item on grabbing. Extreme discrepancies were rare. For example, across the four items on physical discipline, only 0.62% to 6% of mothers and 0.62% to 2% of fathers reported never using the given physical discipline, while their spouse reported that they often used the physical discipline. The reverse, where the children’s mothers or fathers self-reported often using the given physical discipline while their spouse reported they never used the physical discipline, was also rare. However, minor discrepancies were common, leading to slight to moderate agreement between parental self- and spouse-reports (κ ranging from 0.19 to 0.42 across PSDQ items). For example, some children’s mothers or fathers reported never using the given physical discipline while their spouse reported that they used the physical discipline once in a while. It should be noted, however, that minor discrepancies between self- and spouse- reports were comprised of a mix of cases where physical discipline was underreported in self-reports and cases where physical discipline was overreported in self-reports.

**Prevalence of Physical Discipline by Child Sex**

We conducted a series of chi-square tests to examine whether prevalence rates of physical discipline differed by child sex, using parental self-reports of physical discipline at each age of assessment. Table S9 shows the prevalence rates of each physical discipline by child sex at each age of assessment, and the results of the chi-square tests comparing prevalence rates by child sex. The results of the chi-square tests suggest that prevalence rates did not significantly differ by child sex for physical discipline by mothers across the four assessments. Further, prevalence rates did not significantly differ by child sex for spanking, slapping, grabbing, or any form of physical discipline by the father at the age 9 assessment. However, prevalence rates calculated from father’s self-reported general use of physical punishment differed by child sex, such that boys were more likely than girls to be physically punished by their fathers at the age 9 assessment.

**Prevalence of Physical Discipline by Parent Sex**

McNemar’s tests were conducted to examine whether prevalence rates of physical discipline differed by parent sex, using data from 162 children who had both maternal and paternal self-reports of physical discipline in the PSDQ at the age 9 assessment. Table S10 shows the prevalence rates of each physical discipline by mothers and fathers at the age 9 assessment, and the results of the McNemar’s tests comparing prevalence rates by parent sex. The results of the McNemar’s tests suggest that prevalence rates differed significantly by parent sex only for slapping, which was more prevalent among mothers than fathers.

**Prevalence of Maternal Physical Discipline by Maternal Ethnicity**

Chi-square tests were conducted to examine whether prevalence rates of physical discipline by mothers differed by their ethnicity, using maternal self-reports of physical discipline at each age of assessment. Table S11 shows the prevalence rates of each physical discipline by maternal ethnicity at each age of assessment, and the results of the chi-square tests comparing prevalence rates by maternal ethnicity. At the age 4.5, 6, and 9 assessments, the prevalence of slapping significantly differed by maternal ethnicity, with this discipline being most prevalent among Indian mothers. At the age 4.5 assessment, the prevalence of grabbing significantly differed by maternal ethnicity, with the discipline being most prevalent among Indian mothers. At the age 6 assessment, the prevalence of hitting with an object significantly differed by maternal ethnicity, with the discipline being most prevalent among Chinese mothers. Finally, at the age 9 assessment, the prevalence of any form of physical discipline significantly differed by maternal ethnicity, with this prevalence being highest for Malay mothers.

**Change in Prevalence of Physical Discipline of Children from Early to Middle Childhood while Controlling for Children’s Cognitive Skills at Baseline**

Previous literature suggests that parents could rely less on physical discipline as children enter middle childhood and develop the cognitive capabilities to understand reasoning, recognize the consequences of their misbehavior, and control their own behavior.^1,2^  Considering the potential relevance of children’s cognitive skills for the trajectory of parental use of physical discipline, we have rerun our analysis on longitudinal changes in children’s exposure to physical discipline with the inclusion of children’s cognitive skills at age 4.5 years (i.e., baseline age) as covariates. Specifically, we investigated the change in the prevalence of physical discipline from early to middle childhood in 424 children that (a) had maternal reports on their own and the fathers’ use of physical discipline in the PSDQ, and data on age at the time of assessment, in at least one assessment at age 4.5, 9, and 11 years, and (b) completed the Kaufman Brief Intelligence Test, Second Edition (KBIT-2) at age 4.5 years. The KBIT-2 is a brief assessment of verbal and non-verbal intelligence in children aged 4 years to adults aged 90 years, and consists of two subtests (i.e., Verbal Knowledge and Riddles) which yield a verbal score reflective of crystallized abilities and one subtest (i.e., Matrices) which yields a non-verbal score reflective of fluid reasoning.^3^ Children’s verbal and non-verbal scores (i.e., raw scores on relevant subtests) were used for analysis.

We conducted a generalized linear mixed model similar to the one reported in our main analyses, with the addition of children’s verbal and non-verbal intelligence scores at age 4.5 years as covariates. Specifically, a generalized linear mixed model with binomial error structure, logit link function, and maximum likelihood estimation was conducted to examine whether age at each assessment predicts children’s exposure to physical discipline as a binary variable (i.e., 0 = never used, 1 = used once in a while or more frequently). We entered children’s verbal and non-verbal intelligence scores at age 4.5 years, age at each assessment, parent sex (mother or father), physical coercion dimension item (general use of physical punishment, spank, slap, or grab), and the two-way interaction terms of children’s age with parent sex and physical coercion dimension item as fixed effects in the model. Participant ID was included as a random effect variable to account for the repeated measures nature of the data. Findings remain similar to that of our main analysis which did not include children’s cognitive skills as covariates. Specifically, even after controlling for children’s verbal and non-verbal intelligence scores at age 4.5 years, the generalized linear mixed model suggested a significant effect of children’s age, such that children were less likely to be physically disciplined as they became older, (B = -0.14, SE = 0.01, χ^2^(1) = 127.06, *p* < .001, OR = 0.87, 95% CI [0.85, 0.89]). The effects of the interaction of children’s age with parent sex and physical coercion dimension item did not achieve significance (*p*s ≥ .09). We additionally conducted a linear mixed model with restricted maximum likelihood estimation and with the same fixed effects as in the model described above, but with children’s frequency of exposure to physical discipline (i.e., on a Likert scale from 1 = ‘never’ to 5 = ‘always’) as the dependent variable. This model suggested that children’s frequency of exposure to physical discipline decreased with age, even after controlling for children’s verbal and non-verbal intelligence scores at age 4.5 years (B = -0.05, *SE* = 0.004, *F*(1, 7210.13) = 182.94, *p* < .001).

**Relation Between Children’s Exposure to Maternal Physical Discipline at Age 4.5 and 6 and Evaluation of Their Mothers’ Parenting at Age 9**

We conducted simple linear regression analyses to examine whether mothers’ self-reported frequency of using physical discipline when their child was age 4.5 and 6 years would relate to their children’s evaluation of their parenting at age 9 years. Analyses involving maternal physical discipline at age 4.5 years as a predictor were conducted on a subset of 273 children who evaluated their mother in the PBI-C at age 9 and whose mother had completed the PSDQ at age 4.5. Analyses involving maternal physical discipline at age 6 as a predictor were conducted on a subset of 356 children who evaluated their mother in the PBI-C at age 9 and whose mother had completed the APQ at age 6. For the PSDQ, the physical coercion dimension score, or the average of parents’ responses (i.e., 1 to 5) to the four items on physical discipline, were used for analyses. For the APQ, the corporal punishment subscale score, or the sum of parents’ responses (i.e., 1 to 5) to the three items on physical discipline, were used for analyses. For the PBI-C, the sum of children’s responses (i.e., 0 to 3) to items on the mother’s care, denial of psychological autonomy, and denial of behavioral freedom were used for analyses. Holm-Bonferroni adjusted *p* values were calculated to account for three tests for each dimension in the PBI-C.

The regression analyses conducted revealed that mothers’ self-reported frequency of using physical discipline when children were age 4.5 was not related to how children rated their mothers’ parenting, particularly their care (*R*^2^ = .00, *F*(1, 271) = 0.08, B = -0.14, *SE* = 0.50, nominal *p* = .78, adjusted *p* = 1.00), denial of psychological autonomy (*R*^2^ = .00, *F*(1, 271) = 0.44, B = -0.25, *SE* = 0.38, nominal *p* = .51, adjusted *p* = 1.00), and denial of behavioral freedom (*R*^2^ = .01, *F*(1, 271) = 1.45, B = 0.43, *SE* = 0.36, nominal *p* = .23, adjusted *p* = .69), at age 9. Similarly, mothers’ self-reported frequency of using physical discipline when children were age 6 was not related to how children rated their mothers’ care (*R*^2^ = .00, *F*(1, 354) = 0.62, B = -0.12, *SE* = 0.16, nominal *p* = .43, adjusted *p* = 1.00), denial of psychological autonomy (*R*^2^ = .00, *F*(1, 354) = 0.28, B = 0.06, *SE* = 0.12, nominal *p* = .60, adjusted *p* = 1.00), and denial of behavioral freedom (*R*^2^ = .00, *F*(1, 354) = 0.13, B = 0.04, *SE* = 0.11, nominal *p* = .72, adjusted *p* = 1.00) at age 9. We could not investigate how early paternal physical discipline relates to children’s later evaluation of their fathers’ parenting, considering the lack of paternal self-reports at the age 4.5 assessment and limited sample size of fathers (*n* = 44) at the age 6 assessment.

**Parental Physical Discipline in Relation to Parental Warmth and Autonomy Granting**

We used parental reports in the PSDQ at the age 9 assessment to investigate whether parents’ self-reported use of physical discipline was related to their self-reported warmth and autonomy granting towards their child. We were particularly interested in the age 9 assessment to parallel our main analyses examining whether parent’s self-reported use of physical discipline in the PSDQ related to child reports of their parents’ care and denial of psychological autonomy and behavioral freedom at age 9. Pearson’s correlations were conducted to examine whether parents’ physical coercion dimension score, or their average frequency (i.e., 1 = ‘never’ to 5 = ‘always’) for four items on physical discipline, was associated with their (a) connection dimension score, or the average frequency for five items reflecting warmth such as “I am responsive to our child’s feelings or needs” and “I give comfort and understanding when our child is upset”, and (b) autonomy granting dimension score, or the average frequency for five items such as “I take our child’s desires into account before asking the child to do something” and “I take into account our child’s preferences in making plans for the family”. Cronbach’s alphas were 0.71 and 0.82 respectively for maternal and paternal warmth, and 0.67 and 0.76 respectively for maternal and paternal autonomy granting.

We found that mothers’ use of physical discipline was significantly and negatively correlated with their warmth (*r* = -0.13, *p* = .01), but showed no significant correlations with their autonomy granting (*r* = -0.04, *p* = .43). Fathers’ use of physical discipline was significantly and negatively correlated with their warmth (*r* = -0.25, *p* = .001) and their autonomy granting (*r* = -0.17, *p* = .02).

# References

1. Lansford JE, Criss MM, Dodge KA, Shaw DS, Pettit GS, Bates JE. Trajectories of physical discipline: Early childhood antecedents and developmental outcomes. Child Dev. 2009;80(5):1385-1402.

2. Lansford JE, Staples AD, Bates JE, Pettit GS, Dodge KA. Trajectories of mothers’ discipline strategies and interparental conflict: Interrelated change during middle childhood. J Fam Commun. 2013;13(3):178-95.

3. Kaufman AS, Kaufman NL. Kaufman Brief Intelligence Test, second edition*.* Circle Pines, MN: American Guidance Services; 2004.

| **Table S1** |  |  |  |
| --- | --- | --- | --- |
| *Contingency Table for Frequency at Which Children Were Physically Punished by Their Mothers as Reported by Their Mothers and Fathers (n = 162)* | | | |
| Mother’s Self-Reported Use of Physical Punishment | Mother’s Use of Physical Punishment as Reported by Father | | |
|  | Never | Once in a While | Often^a^ |
| Never | 19 | 17 | **1** |
| Once in a While | 18 | 54 | 20 |
| Often^a^ | **3** | 18 | 12 |
| *Note.* Reported frequency in item of physical coercion dimension addressing general use of physical punishment. Percentage agreement = 52.47%, κ = 0.19. Bold font represents strong discrepancy. ^a^Collapsed responses ‘about half of the time’, ‘very often’, and ‘always’. | | | |

| **Table S2** |  |  |  |
| --- | --- | --- | --- |
| *Contingency Table for Frequency at Which Children Were Physically Punished by Their Fathers as Reported by Their Fathers and Mothers (n = 162)* | | | |
| Father’s Self-Reported Use of Physical Punishment | Father’s Use of Physical Punishment as Reported by Mother | | |
|  | Never | Once in a While | Often^a^ |
| Never | 28 | 11 | **1** |
| Once in a While | 30 | 39 | 9 |
| Often^a^ | **5** | 20 | 19 |
| *Note.* Reported frequency in item of physical coercion dimension addressing general use of physical punishment. Percentage agreement = 53.09%, κ = 0.28. Bold font represents strong discrepancy. ^a^Collapsed responses ‘about half of the time’, ‘very often’, and ‘always’. | | | |

| **Table S3** |  |  |  |
| --- | --- | --- | --- |
| *Contingency Table for Frequency at Which Children Were Spanked by Their Mothers as Reported by Their Mothers and Fathers (n = 162)* | | | |
| Mother’s Self-Reported Use of Spanking | Mother’s Use of Spanking as Reported by Father | | |
|  | Never | Once in a While | Often^a^ |
| Never | 27 | 9 | **5** |
| Once in a While | 16 | 47 | 16 |
| Often^a^ | **6** | 17 | 19 |
| *Note.* Reported frequency in item of physical coercion dimension addressing use of spanking. Percentage agreement = 57.41%, κ = 0.33. Bold font represents strong discrepancy. ^a^Collapsed responses ‘about half of the time’, ‘very often’, and ‘always’. | | | |

| **Table S4** |  |  |  |
| --- | --- | --- | --- |
| *Contingency Table for Frequency at Which Children Were Spanked by Their Fathers as Reported by Their Fathers and Mothers (n = 162)* | | | |
| Father’s Self-Reported Use of Spanking | Father’s Use of Spanking as Reported by Mother | | |
|  | Never | Once in a While | Often^a^ |
| Never | 30 | 13 | **3** |
| Once in a While | 27 | 34 | 5 |
| Often^a^ | **9** | 18 | 23 |
| *Note.* Reported frequency in item of physical coercion dimension addressing use of spanking. Percentage agreement = 53.70%, κ = 0.30. Bold font represents strong discrepancy. ^a^Collapsed responses ‘about half of the time’, ‘very often’, and ‘always’. | | | |

| **Table S5** |  |  |  |
| --- | --- | --- | --- |
| *Contingency Table for Frequency at Which Children Were Slapped by Their Mothers as Reported by Their Mothers and Fathers (n = 162)* | | | |
| Mother’s Self-Reported Use of Slapping | Mother’s Use of Slapping as Reported by Father | | |
|  | Never | Once in a While | Often^a^ |
| Never | 78 | 12 | **5** |
| Once in a While | 31 | 20 | 5 |
| Often^a^ | **3** | 3 | 5 |
| *Note.* Reported frequency in item of physical coercion dimension addressing use of slapping. Percentage agreement = 63.58%, κ = 0.29. Bold font represents strong discrepancy. ^a^Collapsed responses ‘about half of the time’, ‘very often’, and ‘always’. | | | |

| **Table S6** |  |  |  |
| --- | --- | --- | --- |
| *Contingency Table for Frequency at Which Children Were Slapped by Their Fathers as Reported by Their Fathers and Mothers (n = 162)* | | | |
| Father’s Self-Reported Use of Slapping | Father’s Use of Slapping as Reported by Mother | | |
|  | Never | Once in a While | Often^a^ |
| Never | 102 | 9 | **1** |
| Once in a While | 15 | 15 | 4 |
| Often^a^ | **4** | 8 | 4 |
| *Note.* Reported frequency in item of physical coercion dimension addressing use of slapping. Percentage agreement = 74.69%, κ = 0.42. Bold font represents strong discrepancy. ^a^Collapsed responses ‘about half of the time’, ‘very often’, and ‘always’. | | | |

| **Table S7** |  |  |  |
| --- | --- | --- | --- |
| *Contingency Table for Frequency at Which Children Were Grabbed by Their Mothers as Reported by Their Mothers and Fathers (n = 162)* | | | |
| Mother’s Self-Reported Use of Grabbing | Mother’s Use of Grabbing as Reported by Father | | |
|  | Never | Once in a While | Often^a^ |
| Never | 55 | 15 | **10** |
| Once in a While | 23 | 27 | 8 |
| Often^a^ | **6** | 10 | 8 |
| *Note.* Reported frequency in item of physical coercion dimension addressing use of grabbing. Percentage agreement = 55.56%, κ = 0.27. Bold font represents strong discrepancy. ^a^Collapsed responses ‘about half of the time’, ‘very often’, and ‘always’. | | | |

| **Table S8** |  |  |  |
| --- | --- | --- | --- |
| *Contingency Table for Frequency at Which Children Were Grabbed by Their Fathers as Reported by Their Fathers and Mothers (n = 162)* | | | |
| Father’s Self-Reported Use of Grabbing | Father’s Use of Grabbing as Reported by Mother | | |
|  | Never | Once in a While | Often^a^ |
| Never | 60 | 15 | **2** |
| Once in a While | 20 | 22 | 9 |
| Often^a^ | **15** | 7 | 12 |
| *Note.* Reported frequency in item of physical coercion dimension addressing use of grabbing. Percentage agreement = 58.02%, κ = 0.31. Bold font represents strong discrepancy. ^a^Collapsed responses ‘about half of the time’, ‘very often’, and ‘always’. | | | |

| **Table S9** | | | | | | |  | |
| --- | --- | --- | --- | --- | --- | --- | --- | --- |
| *Prevalence of Physical Discipline of Children by Their Parents at Age 4.5, 6, 9, and 11 According to Child Sex* | | | | | | | | |
| Age of Assessment (Questionnaire)^a^ | Parent | *n* | Physical Discipline in Subscale | Child Sex | | | | χ^2^ |
|  |  |  |  | Girls^b^ |  | Boys^b^ | |  |
| Age 4.5 (PSDQ) | Mother | Girls *n* = 192 Boys *n* = 207 | Physical Punishment, General % | 82 |  | 86 | | 0.76 |
|  |  |  | Spank % | 80 |  | 82 | | 0.38 |
|  |  |  | Slap % | 45 |  | 42 | | 0.58 |
|  |  |  | Grab % | 58 |  | 58 | | 0.00 |
|  |  |  | **Any Form of Physical Discipline %^c^** | **96** |  | **93** | | 1.29 |
| Age 6 (APQ)^d^ | Mother | Girls *n* = 259 Boys *n* = 279 | Spank % | 88 |  | 92 | | 1.69 |
|  |  |  | Slap % | 56 |  | 54 | | 0.19 |
|  |  |  | Hit With Object % | 43 |  | 49 | | 2.09 |
|  |  |  | **Any Form of Physical Discipline %^c^** | **92** |  | **94** | | 0.83 |
| Age 9 (PSDQ) | Mother | Girls *n* = 200 Boys *n* = 214 | Physical Punishment, General % | 76 |  | 79 | | 0.71 |
|  |  |  | Spank % | 70 |  | 76 | | 2.33 |
|  |  |  | Slap % | 38 |  | 40 | | 0.13 |
|  |  |  | Grab % | 49 |  | 51 | | 0.15 |
|  |  |  | **Any Form of Physical Discipline %^c^** | **88** |  | **89** | | 0.31 |
|  | Father | Girls *n* = 82 Boys *n* = 86 | Physical Punishment, General % | 70 |  | 83 | | 3.94^*^ |
|  |  |  | Spank % | 70 |  | 76 | | 0.78 |
|  |  |  | Slap % | 27 |  | 36 | | 1.65 |
|  |  |  | Grab % | 50 |  | 56 | | 0.57 |
|  |  |  | **Any Form of Physical Discipline %^c^** | **83** |  | **91** | | 2.23 |
| Age 11 (PSDQ) | Mother | Girls *n* = 190 Boys *n* = 203 | Physical Punishment, General % | 68 |  | 67 | | 0.04 |
|  |  |  | Spank % | 67 |  | 67 | | 0.01 |
|  |  |  | Slap % | 28 |  | 31 | | 0.21 |
|  |  |  | Grab % | 43 |  | 41 | | 0.13 |
|  |  |  | **Any Form of Physical Discipline %^c^** | **82** |  | **80** | | 0.21 |
| *Note. *p* < .05. ^a^Data from the physical coercion dimension of the PSDQ at the age 4.5, 9, and 11 assessments, and from the corporal punishment subscale of the APQ at the age 6 assessment. ^b^Percentage of girls and boys whose parents endorsed using the physical discipline, regardless of frequency of use*.* ^c^In bold font are the percentages of girls and boys whose parents, regardless of frequency, endorsed using at least at one physical discipline. ^d^Paternal self-reports were not analyzed due to the small sample size (*n* = 44). | | | | | | | | |

| **Table S10** | | |  |
| --- | --- | --- | --- |
| *Prevalence of Physical Discipline of Children by Their Mothers and Fathers at Age 9* (*n* = 162) | | | |
| Physical Coercion Dimension Item | Parent Sex | | χ^2^ |
|  | Mother | Father |  |
| Physical Punishment, General %^a^ | 77 | 75 | 0.21 |
| Spank %^a^ | 75 | 72 | 0.49 |
| Slap %^a^ | 41 | 31 | 5.25^*^ |
| Grab %^a^ | 51 | 52 | 0.15 |
| **Any Form of Physical Discipline %^b^** | **88** | **86** | 0.29 |
| *Note.* ^*^*p* < .05. An exploratory generalized linear mixed model with binomial error structure and logit link function suggested no significant interaction between parent sex and child sex on whether children were exposed to physical discipline at any frequency. ^a^Percentage of children whose parents endorsed using the physical discipline, regardless of frequency of use. ^b^In bold font are the percentages of children whose parents, regardless of frequency, endorsed using at least one physical discipline. | | | |

| **Table S11** | | | | | |  |  |  |
| --- | --- | --- | --- | --- | --- | --- | --- | --- |
| *Prevalence of Physical Discipline of Children by Their Mothers at Age 4.5, 6, 9, and 11 by Maternal Ethnicity* | | | | | | | | |
| Age of Assessment (Questionnaire)^a^ | *n* | Physical Discipline in Subscale | Maternal Ethnicity | | | | | χ^2^ |
|  |  |  | Chinese^b^ |  | Malay^b^ |  | Indian^b^ |  |
| Age 4.5 (PSDQ) | Chinese *n* = 229 Malay *n* = 109 Indian *n* = 60 | Physical Punishment, General % | 87 |  | 82 |  | 77 | 4.78 |
|  |  | Spank % | 78 |  | 84 |  | 85 | 2.63 |
|  |  | Slap % | 39 |  | 44 |  | 58 | 7.38^*^ |
|  |  | Grab % | 53 |  | 63 |  | 68 | 6.39^*^ |
|  |  | **Any Form of Physical Discipline %^c^** | **94** |  | **94** |  | **97** | 0.73 |
| Age 6 (APQ) | Chinese *n* = 307 Malay *n* = 149 Indian *n* = 81 | Spank % | 90 |  | 91 |  | 90 | 0.33 |
|  |  | Slap % | 51 |  | 55 |  | 73 | 12.58^**^ |
|  |  | Hit With Object % | 54 |  | 37 |  | 36 | 16.30^***^ |
|  |  | **Any Form of Physical Discipline %^c^** | **92** |  | **93** |  | **95** | 0.85 |
| Age 9 (PSDQ) | Chinese *n* = 229 Malay *n* = 120 Indian *n* = 64 | Physical Punishment, General % | 76 |  | 82 |  | 75 | 1.89 |
|  |  | Spank % | 72 |  | 78 |  | 66 | 3.59 |
|  |  | Slap % | 34 |  | 39 |  | 58 | 12.31^**^ |
|  |  | Grab % | 48 |  | 52 |  | 55 | 1.05 |
|  |  | **Any Form of Physical Discipline %^c^** | **85** |  | **94** |  | **91** | 7.22^*^ |
| Age 11 (PSDQ) | Chinese *n* = 245  Malay *n* = 105 Indian *n* = 42 | Physical Punishment, General % | 69 |  | 67 |  | 64 | 0.57 |
|  |  | Spank % | 67 |  | 66 |  | 69 | 0.15 |
|  |  | Slap % | 27 |  | 31 |  | 38 | 2.22 |
|  |  | Grab % | 38 |  | 47 |  | 55 | 5.04 |
|  |  | **Any Form of Physical Discipline %^c^** | **82** |  | **77** |  | **86** | 1.95 |
| *Note. ^*^p* < .05, *^**^p* < .01, *^***^p* < .001*.* ^a^Data from the physical coercion dimension of the PSDQ at the age 4.5, 9, and 11 assessments, and from the corporal punishment subscale of the APQ at the age 6 assessment. ^b^Percentage of children, by maternal ethnicity, whose mothers endorsed using the physical discipline, regardless of frequency of use. One participant was excluded across the four assessments due to belonging to none of the three major ethnic groups in Singapore*.* ^c^In bold font are the percentages of children, by maternal ethnicity, whose mothers endorsed using at least one physical discipline, regardless of frequency of use. | | | | | | | | |
